# The Exercise-Induced Irisin Is Associated with Improved Levels of Glucose Homeostasis Markers in Pregnant Women Participating in 8-Week Prenatal Group Fitness Program: A Pilot Study

**DOI:** 10.1155/2017/9414525

**Published:** 2017-10-31

**Authors:** Anna Szumilewicz, Aneta Worska, Magdalena Piernicka, Agnieszka Kuchta, Jakub Kortas, Zbigniew Jastrzębski, Łukasz Radzimiński, Joanna Jaworska, Katarzyna Micielska, Ewa Ziemann

**Affiliations:** ^1^Department of Fitness and Strength Conditioning, Gdansk University of Physical Education and Sport, Gdansk, Poland; ^2^Department of Clinical Chemistry, Medical University of Gdansk, Gdansk, Poland; ^3^Department of Recreation and Qualified Tourism, Gdansk University of Physical Education and Sport, Gdansk, Poland; ^4^Department of Biomedical Health Basics, Gdansk University of Physical Education and Sport, Gdansk, Poland; ^5^Department of Physiology and Pharmacology, Gdansk University of Physical Education and Sport, Gdansk, Poland; ^6^Department of Anatomy and Anthropology, Gdansk University of Physical Education and Sport, Gdansk, Poland

## Abstract

**Background:**

Both exercise and pregnancy influence serum irisin concentration.

**Aim:**

To determine how the interaction of pregnancy and exercise affects irisin level and whether various patterns of exercise adherence had different effect on irisin concentration.

**Methods:**

It was a one-group pretest-posttest study among 9 Caucasian nulliparous healthy women in normal pregnancy (age 23 ± 3 years, 21 ± 2 weeks of gestation; mean ± SD) who participated in 8-week group fitness program. Before and after exercise intervention, we determined serum concentrations of irisin and selected parameters of lipid profile and glucose homeostasis markers.

**Results:**

In active women, irisin slightly decreased with the development of pregnancy. After 8 weeks of exercising, irisin correlated negatively with fasting glucose (*R* = −0.922; *p* = 0.001), glycated hemoglobin (*R* = −0.784; *p* = 0.012), and insulin concentrations (*R* = −0.845; *p* = 0.004). In women exercising below recommended level, we observed a significant drop in irisin concentration, whereas in women exercising at least three times a week this myokine slightly increased (31% difference; 90% confidence limits ±28; a large, clear effect).

**Conclusions:**

Irisin stimulated by prenatal exercise may improve glucose homeostasis markers in healthy women and compensate for metabolic changes induced by pregnancy. Moreover, the frequency of exercise may regulate the changes in exercise-induced irisin concentration.

## 1. Introduction

Although pregnant women should perform at least 150 min of moderate-intensity aerobic activity per week [1], only 15% of them adhere to these guidelines [2]. In pregnancy, physical inactivity and excessive weight gain have been recognized as independent risk factors for maternal obesity and related pregnancy complications, including gestational diabetes mellitus (GDM) [1, 3]. Healthy pregnancy can be associated with resistance to the action of insulin on glucose uptake and utilization. This leads to more use of fats than carbohydrates for energy by mother and saves carbohydrates for the growing fetus [4]. In 1–14% of pregnant women, this condition develops into GDM [5], which increases the risk of macrosomia, birth complications, and maternal diabetes after pregnancy. It may also increase the risk of obesity and type 2 diabetes in offspring later in life [6]. Thus, any strategy to prevent GDM should be considered.

Garces and coworkers [7] suggest that irisin concentration is significantly related to the changing insulin sensitivity in healthy pregnant women, regardless of the trimester of gestation and other variables. Irisin is an exercise-inducible myokine that regulates the differentiation of adipose tissue, increasing the energy expenditure and reducing weight and insulin resistance [8]. In addition, irisin activates oxygen consumption in white fat cells and thermogenesis [9]. Irisin protein expression in placenta is low and, therefore, probably not a major contributor to serum concentrations and potential effects of the myokine in pregnancy [10]. Higher irisin concentration was noted in middle and late pregnancy compared to early pregnancy in healthy women, with an increase of approximately 16% and 21%, respectively [7]. Kuzmicki et al. also observed that serum irisin increases markedly in pregnant women, but this increase seems to be significantly lower in patients with GDM [11]. The study by Ural et al. [12] supports these data. Thereby irisin may be a useful biomarker in early pregnancy to predict the development of GDM [13].

Rodrigues et al. [14] have presented a systematic review on how the intensity, duration, and type of exercise can affect the serum irisin concentration in healthy adults, both in men and in nonpregnant women. However, data concerning the influence of physical activity on irisin levels during pregnancy has not been published so far. Thus in this study, we aimed to determine how the interaction of uncomplicated pregnancy and structured exercise program affects serum irisin. The second objective of the study was to answer whether various patterns of exercise adherence had different effect on this myokine concentrations.

## 2. Methods

**Figure 1:**
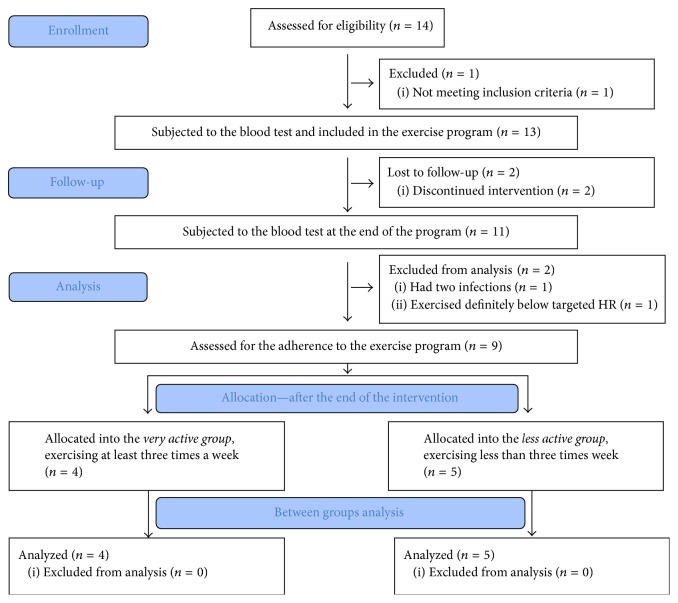
The flow of participants through the study.

The design was a one-group pretest-posttest study among 9 Caucasian nulliparous healthy women in normal pregnancy (age 23 ± 3 years, 21 ± 2 weeks of gestation; mean ± SD). All women volunteered for the study by completing an electronic form available on the web site of the experiment. The eligibility criteria were a positive assessment of woman's health and normal pregnancy confirmed by an obstetric care provider, single pregnancy, and meeting recommended level of physical activity between the conception and the beginning of the experiment. Women's health condition and the course of pregnancy were assessed on the routine medical consultation, according to the national law. The level of physical activity before the experiment was assessed using the Pregnancy Physical Activity Questionnaire [15]. Exclusion criterion was history of miscarriages over 12 weeks of gestation and/or more than two successive miscarriages in the first trimester. The flow of participants through the study is presented in Figure 1.

We conducted the study in Laboratory of Physical Effort and Genetics in Sport, at Gdansk University of Physical Education and Sport (AWFiS) in Poland between January and March 2016. It was performed according to the principles of the Helsinki Declaration and the project's approval of the Bioethics Commission in Gdansk (KB 8/13 and KB 22/15). The participants signed the informed consent before testing.

### 2.1. Assessment of the Exercise Capacity

At the beginning of exercise program, all women underwent an exercise test on a cycloergometer with electronically regulated load (Viasprint 150P). In order to establish the maximum oxygen uptake we have used stationary respiratory gas analyzer (Oxycon Pro, Erich JAEGER GmbH, Germany). It was calibrated prior to each test according to the manufacturer's instructions. Breath-by-breath data were averaged to provide a data point for each 15-second period.

The test started with a 5-minute adaptation phase when women sat on a chair. There followed a 4-minute warm-up with relative load of 0.4 W·kg^−1^ of body mass. After the warm-up, the load increased by 0.2 W·kg^−1^ every minute, up to refusal. Before the experiment we instructed women to use the 0–10 Borg's Perceived Exertion Scale [16]. They were allowed to stop the test at any time. As women's maximal effort, we treated the test results when they achieved the perceived exertion level of 9 or 10 and the value of Respiratory Exchange Ratio (RER) was above 1. Women were cycling during the test for an average of 16 ± 2 min. After the test, the participants rested for 3 minutes sitting on a chair. The highest oxygen uptake achieved during the maximum effort and maintained for 15 seconds was taken as maximal oxygen capacity (VO_2 max_).

Based on the RER value we had set heart rate zones for exercise sessions. The lower heart rate limit corresponded to the RER value of 0.85. Above this intensity carbohydrates usually start to be predominant source in energy yielding in response to exercise [17]. The upper heart rate limit was set at the RER value equal to 1, which corresponds to the maximal lactate steady state (MLSS) [18]. MLSS represents the exercise intensity above which a continuous increase in blood lactate is unavoidable and refers to the term “anaerobic threshold” [19]. Keeping heart rate between these thresholds ensured that participants performed aerobic exercises and optimize cardiopulmonary fitness [17]. Aerobic physical activity, apart from numerous benefits typical for general populations, compensates for the physiological changes in woman's body induced by pregnancy [20]. It is also considered that it safely helps to control glycemic homeostasis during gestation [21].

### 2.2. Blood Collection and Analysis

Before the first exercise session, blood samples were taken from the antecubital vein into the vacutainer tubes with EDTAK_2_ by a professional nurse_._ For the purpose of assessing glucose level blood was taken into the vacutainer tubes with sodium fluoride. In order to control glucose alternation, glycated hemoglobin was also determined. After the blood collection, all women ate the same light breakfast and after 50–60 minutes of rest they started to exercise. The same schedule of blood collection was applied before the last exercise session at the end of 8-week exercise program. At both time points, the blood samples were taken in a fasting condition. We asked participants to follow recommendations for proper nutrition during pregnancy as well as not to introduce any changes in diet or additional supplements during the experiment.

Immediately following the blood collection, one portion of the sample was transferred to centrifuge tubes containing aprotinin (catalog number RK-APRO) from Phoenix Pharmaceuticals Inc. The final concentration of aprotinin was 0.6 Trypsin Inhibitor Unit/1 ml of blood. The samples were centrifuged at 2000 g for 10 min at 4°C. The separated plasma samples were frozen and kept at –70°C until later analysis. Quantification of plasma irisin was based on a competitive enzyme immunoassay and the assay kits were purchased from Phoenix Pharmaceuticals Inc. (catalog number EK 067-16). Details of the ELISA assay have been described elsewhere [22]. The dilution of sample was 1 : 5. The intra-assay coefficients of variability (CVs) and interassay CVs reported by the manufacturer were 4%–6% and 8%–10%, respectively. Due to many doubts in assessing irisin we used the one of a recommended commercial kit [23].

Insulin was determined also by enzyme immunoassay methods using commercial kit DiaMetra (DCM076-8). The within assay variability was ≤5%. The hematological measurements were performed using conventional methods with a Coulter® LH 750 Hematology Analyzer (Beckman-Coulter, USA). Glucose was assessed using analyzer Cobos 6000. The serum concentrations of the total cholesterol (TC), high and low density-lipoproteins (HDL, LDL), and triglycerides (TG) were determined with commercial kits using enzymatic methods (Alpha Diagnostics, Poland).

### 2.3. Prenatal Exercise Program as Experimental Intervention

Pregnant women participated in the 8-week structured exercise program, designed by the principal researcher of the study according to the available guidelines [1, 24]. Group exercise sessions were held three times a week on Mondays, Wednesdays, and Fridays from 9.30–10.30 a.m. at the sport facilities of Gdansk University of Physical Education and Sport. Full attendance in the program provided women with recommended level of physical activity of at least 150 minutes per week.

Each session consisted of warm-up and aerobic part in the form of high-low impact fitness choreography with music (25 min), strength-conditioning exercises (25 min), stretching and breathing exercises, and relaxation (10 min). To maintain proper intensity of exercise during aerobic part we used heart rate monitors (Polar RS400, Finland) in each session with individually adjusted heart rate zones. We trained women how to observe changes in their heart rate and to keep it within the stated ranges. Additionally, they monitored the exercise intensity based on the “talk test” and the Rating of Perceived Exertion (RPE) scale [1]. In the strengthening part women performed nine exercises for each muscle group in two sets of 12–16 repetitions, with a break of 30 s between sets. We instructed the participants to perform the repetitions until they felt unpleasant soreness of the targeted muscles. No equipment was used during exercises and only resistance of own body was applied.

The sessions were conducted by a certified Pregnancy and Postnatal Exercise Specialist whose competences met the European educational standard for this profession [25]. She was informed of the aim of the study and trained in terms of monitoring and maintaining the desired intensity of exercise among participants (inter alia by using rest breaks or implementing jumps and optional repetitions). The principal researcher was checking the quality of exercise program implementation once every two weeks. We used email and phone contact to keep the adherence to the program. The exercise specialist checked and registered attendance for each session. She also recorded reasons for absence and/or additional physical activity performed by the participants individually between sessions. Women were supposed to maintain the same intensity of exercise also in the individual activities, using “talk test” and RPE scale.

### 2.4. Statistical Analysis

Classical statistical analysis was performed using the Statistica software package (Statistica 10.0 Statsoft Poland) and Graphpad Prism 4.03 software. Continuous variables were expressed as mean ± standard deviation (SD). Univariate correlations were assessed using standardized Spearman coefficients. The *p* value obtained of less than 0.05 was considered statistically significant.

For more in-depth analysis, after the experiment, we assessed women for the adherence to the exercise program and allocated them into two groups (Table 1). Among nine participants of the study four pregnant women participated in exercise sessions at least three times a week (very active group; completed sessions 31 ± 4; mean ± SD). Five pregnant women exercised below recommendations, less than 3 times a week (less active group; completed sessions 20 ± 3; mean ± SD).

Due to the small size of the groups and insufficient power of classical test, all measures were compiled in Hopkins'* pre-post parallel-groups trial spreadsheet* [26]. All data were log-converted to reduce bias arising from error nonuniformity. Probabilistic conclusions about the true (large-sample) value of effects were provided in the spreadsheet as magnitude-based inferences [27]. We expressed uncertainty in each effect as 90% confidence limits and as probabilities that the true effect was beneficial (e.g., a substantial increase in the irisin level) and harmful (e.g., a substantial decrease in the in the irisin level). Clinically clear beneficial effects were those for which benefit was at least possible (>25% chance) and risk of harm was acceptably low (<0.5%). Effects where chance of benefit outweighed risk of harm (an odds ratio of benefit to harm > 66) were also deemed clear. Other effects were either clearly nonbeneficial (chance of benefit < 25%) or unclear (chance of benefit > 25% and risk of harm > 0.5%). Clear effects were reported as the magnitude of the observed value, with the qualitative probability that the true effect was beneficial, trivial, or harmful for the change (e.g., in the irisin level). The scale for interpreting the probabilities was as follows: 25–75%, possible; 75–95%, likely; 95–99.5%, very likely; >99.5%, most likely [27]. As a threshold value for the smallest important or harmful effect we used 0.2. Because the recommended level of prenatal physical activity is a minimum of 150 min per week, as reference values (control group in Hopkins' spreadsheet) we treated the results of women who met these guidelines (very active group).

## 3. Results

In Table 1, we presented the characteristics of the whole study group as well as the subgroups separated by the attendance rate. Very active and less active groups in terms of age, week of gestation, BMI, physical fitness, and exercise heart rate zones presented similar values (Table 1).

In all physically active women we recorded 14.78 ± 3.47 ng·ml^−1^ and 14.28 ± 4.39 ng·ml^−1^ (M ± SD) of circulating irisin in 21st and 29th week of gestation, respectively (Table 2). Other selected blood parameters before and after eight weeks of exercise program corresponded to the reference values for pregnancy [28].

**Figure 2:**
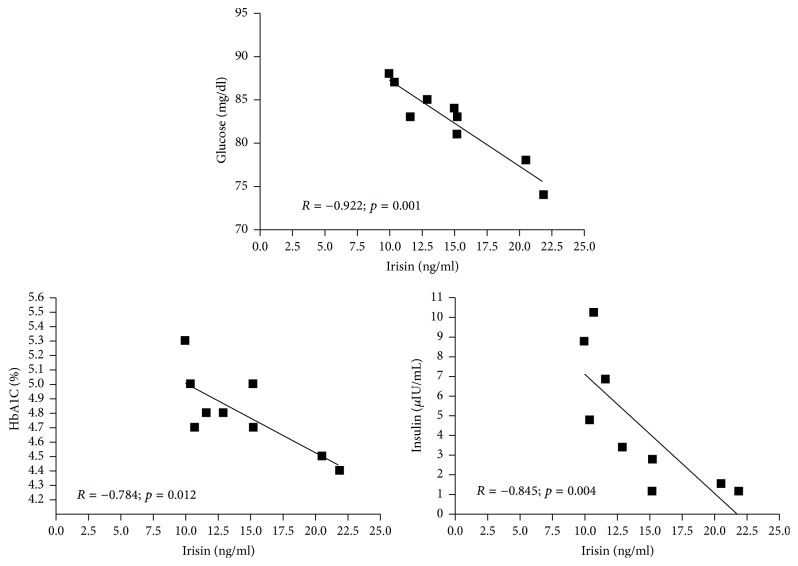
Correlations between irisin concentration and glucose homeostasis markers in women (*n* = 9) in 29th week of gestation after 8 weeks of exercise program.

**Figure 3:**
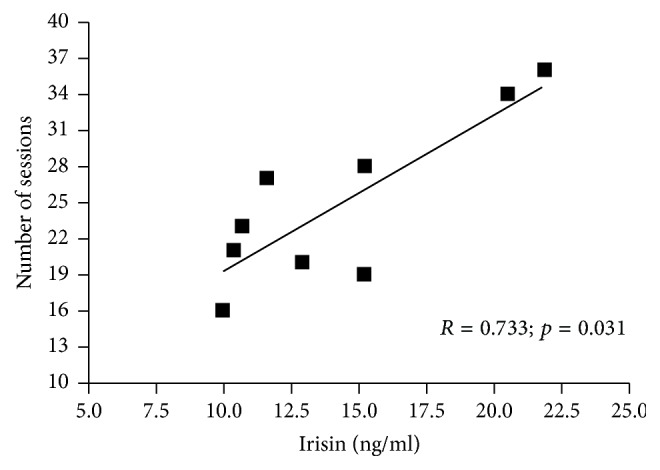
Correlations between irisin concentration and number of exercise sessions performed by pregnant women (*n* = 9) during 8 weeks of exercise program.

Before exercise intervention (21st week of gestation) we found no relationships between irisin concentration and glucose homeostasis markers or lipid profile (Table 3). However after 8 weeks of exercising (29th week of gestation) irisin levels correlated negatively with fasting glucose, glycated hemoglobin, and insulin concentrations (Figure 2). We observed positive association between irisin level and number of exercise sessions performed by pregnant women during 8 weeks of exercise program (Figure 3).

**Figure 4:**
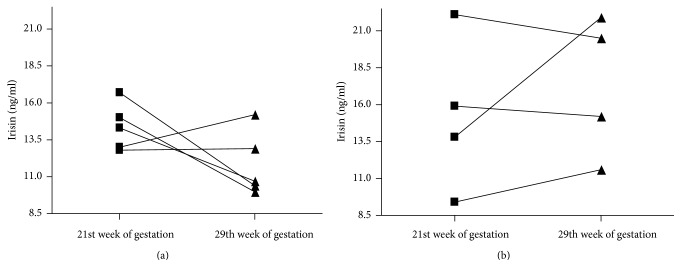
The irisin concentration before and after 8-week exercise program (21st and 29th week of gestation, resp.) in women who had physical activity below recommendations ((a) less active group) and in women who exercised at least three times a week ((b) very active group).

The irisin concentrations before the exercise program (21st week of gestation) in very active and less active groups were 15.32 ± 5.3 and 14.3 ± 1.5 ng·ml^−1^, respectively. After 8 weeks in three participants exercising below the recommended level we observed a significant decrease in irisin concentration (average by 32%; min 31%, max 36%). We did not record similar reductions in irisin level in women who exercised at least three times a week. In this study group, the maximum decrease in the level of irisin was 4%, and the two participants had an irisin increase of 24 and 58% (Figure 4).

Changes in lipid profile and glucose homeostasis markers in response to exercise intervention in women from very active and less active groups are presented in Table 4. Comparing the mean change in the pre- and posttraining irisin level between groups using magnitude-based inferences we observed a large, clear effect. The irisin level was substantially lower in women who exercised below recommendations relative to women exercising at least three times a week.

## 4. Discussion 

To the best of our knowledge, this is the first study determining irisin levels in pregnant women participating in a structured exercise program.

The unexpected result of our study is that the serum concentration of irisin slightly decreased with the development of pregnancy in contrast to the findings presented in other studies [7, 11, 13]. Some authors hypothesized that elevated circulating irisin is an adaptive response to compensate for the increasing insulin resistance and limit the adverse metabolic and vascular effects of pregnancy [10, 13]. Ebert et al. [29] observed that homeostasis model assessment of insulin resistance (HOMA-IR) remains as a positive predictor of irisin serum concentrations. It should be underlined that physical activity of moderate to high intensity significantly decreases insulin resistance in pregnant women [30]. We can assume that physical activity removes the potential cause for higher secretion of irisin. This can explain very similar irisin level in 21st and 29th week of gestation in regularly exercising pregnant women.

In nonpregnant healthy subjects circulating irisin levels are associated with a beneficial metabolic profile [29]. In pregnancy the interaction of circulating irisin and metabolic parameters seems to change significantly. In the study by Piya et al. [31] serum irisin in the pregnant women was positively correlated with glucose, insulin, HOMA-IR, total cholesterol, TG, LDL, and HDL. Ebert et al. [10] also found positive correlation between irisin and fasting insulin, HOMA-IR, and total cholesterol in healthy pregnant women. Among participants of our experiment, we did not record correlation between irisin, lipid profile, and glucose concentration at baseline. However, after 8 weeks of group fitness program (29th week of gestation) irisin levels inversely correlated with fasting glucose, glycated hemoglobin, and insulin concentrations. Our results might suggest that irisin stimulated by exercise leads to improvement in the levels of glucose homeostasis markers and may compensate for metabolic changes induced by pregnancy. Recently published review summarized the particular role of irisin in glucose homeostasis. Available data indicated that the elevated concentration of irisin enhanced glucose and fatty acid uptake (by 30–40%). This increase in glucose uptake results from the upregulation of glucose transporter type 4 (GLUT4) expression, without significant changes in the expression genes encoding insulin receptors [23]. Observed correlations in our group of women might confirm that information. Moreover the drop of insulin accompanied by the increase of irisin may reveal the improvement of muscle insulin sensitivity.

An interesting question is why we did not observe significant relationship between irisin and serum lipids in our participants. We can hypothesize that the implemented prenatal exercise program had more effect on glucose than lipid metabolism due to the assumed intensity of physical exertion. Women performed exercise with the intensity of HR corresponding to RER value between 0.85 and 1.0, which means that carbohydrates were the predominant source in energy yielding by the muscle [17]. It would be valuable to observe the irisin level and its relationship to serum lipids in pregnant women exercising with lower intensity leading to greater fat utilization in future research.

Interestingly, we observed strong positive correlation between irisin and the number of exercise sessions performed by the study participants. The influence of physical activity on irisin still is not clear. Regular exercise affects the irisin concentration in both man and women. In the study by Zhao at al. [32], after 12-week resistance exercise program, the circulating irisin was significantly elevated in the older male adults. Similarly, Kim et al. [33] observed higher serum concentrations of irisin in the trained group of nonpregnant women compared to the control group after 12 weeks of resistance training. However, due to potentially gender dimorphism of irisin [34] which probably relates to female reproductive function [7], pregnant women may respond differently to exercise regarding this myokine secretion. Until now no data are available on how prenatal physical activity affects irisin concentration. Exercise program implemented in our experiment consisted of aerobic part and strength-conditioning exercises, so two different training stimuli could have affected irisin production. In future studies it would be interesting to compare irisin levels in pregnant women undergoing various types of physical exertion.

In order to plan an effective prenatal training program, the type, intensity, frequency, and duration of exercise sessions should be appropriately adjusted to the physiological and biomechanical needs of the pregnant [24]. In this experiment, we found substantial differences in irisin, possibly determined by the frequency of exercise. In less active group (exercising less than three times a week) the exercise program induced a clear drop, whereas in very active group (exercising at least three times a week) a slight increase in irisin was noted. These different responses to exercise may be related to different total training loads during 8 weeks of the experiment resulting from different total volume of exercise during program (duration of each session multiplied by the number of sessions performed).

On the other hand, differences in irisin may depend on previous physical activity patterns. Although both groups met the same inclusion criteria and presented the similar level of various parameters at baseline, it is probable that women who could not keep the recommended exercise regime during the experiment had been less active also before its implementation. Another interpretation for our results is related to the source of irisin. Roca-Rivada and coworkers showed that both human subcutaneous and visceral fat tissue express and secret FNDC5/irisin, which indicates that irisin may be also adipokine [35]. Moreover, Dulian and coworkers also observed the rise of irisin concentration in obese men in response to low temperatures [36]. They noted correlations between body composition and irisin concentration, suggesting that subcutaneous fat tissue, rather than skeletal muscle, was the main source of irisin. To use these findings to interpret our observations in pregnant women it would be necessary to thoroughly analyze their body composition. It is also tempting to speculate that in relation to irisin there is a threshold for training load in which physical activity not only compensates for the pregnancy-induced metabolic changes, but likewise gives a posttraining supercompensation effect. Clearly, these hypotheses need to be tested in future studies.

An important finding is that among our participants the decreasing irisin was not associated with complications during pregnancy, as the results of previous studies would suggest. Other authors observed that in women with gestational diabetes mellitus the irisin concentration was lower than in healthy subjects [10–12]. In our study group of physically active pregnant women we have not reported any case of GDM or preeclampsia.

Obviously, the weakest point of our study is the small number of the participants, which definitely limits the possibility of generalizations. For ethical reasons we decided to conduct a quasi-experimental study instead of randomized control trial [37]. At least 150 minutes per week of physical activity has been recommended for uncomplicated course of pregnancy [1]. It would be unethical to randomize pregnant women to the intervention of lower physical activity level or encourage them to physical inactivity. We can assume, however, that a comparison of changes between groups of physically active and inactive pregnant females would give more varied results in serum irisin concentrations and other parameters.

Another limitation is that we could not refer our observations to the findings by other authors on irisin levels in pregnancy, as they have not presented data on the physical activity levels of their subjects.

## 5. Conclusions

In healthy, physically active women with uncomplicated pregnancy, the serum irisin concentration slightly decreased between the second and the third trimester. These observations contradict the results of other authors, who found that the irisin level elevates markedly with the development of pregnancy. However, pregnant women in other studies did not participate in regular physical activity. Therefore, we can conclude that the structured, prenatal exercise programs may compensate for metabolic changes induced by pregnancy, also those related to increased irisin secretion. Higher irisin concentration in serum was related to better glucose homeostasis.

The frequency of exercise substantially differentiated the level of this myokine in pregnant women.

Our results support the promotion of physical activity during pregnancy and educational activities for the obstetric care providers and exercise specialists enabling them to implement well-designed prenatal exercise programs, meeting different needs of pregnant exercisers. Because of the health benefits, pregnant women should be encouraged to fulfill the recommended level of physical activity.

## Figures and Tables

**Table 1:** Characteristics of the study participants.

Variable at baseline	All pregnant women *n* = 9 (M ± SD)	Very active group^1^ *n* = 4 (M ± SD)	Less active group^2^ *n* = 5 (M ± SD)
Age, y	29 ± 3	29 ± 4	29 ± 3
Gestational age, wk	21 ± 3	22 ± 2	21 ± 3
BMI, kg·m^−2^	22.5 ± 2.5	21.8 ± 3.0	23.0 ± 2.2
VO_2 max_, ml·kg^−1^·min^−1^	23 ± 5.0	23.5 ± 6.6	22 ± 4.1
HR zones for exercise sessions			
HR lower limit (b·min^−1^)	121 ± 12	121 ± 15	121 ± 12
HR upper limit (b·min^−1^)	143 ± 12	141 ± 15	145 ± 11

^1^Participated in exercise sessions at least three times a week; ^2^physically active below recommendations, less than 3 times a week; BMI: body mass index; VO_2 max_: maximal oxygen capacity; HR: heart rate.

**Table 2:** Selected blood parameters in pregnant women (*n* = 9) before and after exercise program.

Variable	Before exercise program 21st week of gestation	After exercise program 29th week of gestation	Post-pre Difference in mean
	Mean ± SD	Mean ± SD	%
Irisin (ng·ml^−1^)	14.78 ± 3.47	14.28 ± 4.39	−3
Glucose (mg·dl^−1^)	80.78 ± 4.27	82.38 ± 4.66	2
HbA1c (%)	4.71 ± 0.28	4.80 ± 0.27	2
Insulin (*µ*lU·ml^−1^)	3.05 ± 1.82	4.51 ± 3.39	48
TG (mg·dl^−1^)	123 ± 36.47	187.88 ± 65.15	53
TC (mg·dl^−1^)	232.89 ± 50.22	266.56 ± 34.81	14
LDL (mg·dl^−1^)	120.56 ± 38.8	148.67 ± 31.50	23
HDL (mg·dl^−1^)	88.11 ± 18	82.00 ± 15.27	−7

HbA1c: glycated hemoglobin; TG: triglycerides; TC: total cholesterol; LDL: low density-lipoproteins; HDL: high density-lipoproteins.

**Table 3:** Correlations between irisin, lipids, and glucose homeostasis markers in physically active pregnant women (*n* = 9).

Irisin (ng·ml^−1^)	Before exercise program	After exercise program
	21st week of gestation	29th week of gestation
Glucose (mg·dl^−1^)	*R* = −0.068; *p* = 0.861	*R* = −0.922; *p* = 0.001^*∗*^
HbA1c (%)	*R* = 0.093; *p* = 0.811	*R* = −0.784; *p* = 0.012^*∗*^
Insulin (*µ*lU·ml^−1^)	*R* = 0.166; *p* = 0.668	*R* = −0.845; *p* = 0.004^*∗*^
TG (mg·dl^−1^)	*R* = −0.161; *p* = 0.460	*R* = −0.503; *p* = 0.204
TC (mg·dl^−1^)	*R* = −0.500; *p* = 0.170	*R* = −0.385; *p* = 0.306
LDL (mg·dl^−1^)	*R* = −0.617; *p* = 0.077	*R* = −0.300; *p* = 0.432
HDL (mg·dl^−1^)	*R* = −0.083; *p* = 0.831	*R* = 0.250; *p* = 0.516

Univariate correlations were assessed using standardized Spearman coefficients; ^*∗*^the *p* value obtained of less than 0.05 was considered statistically significant; HbA1c: glycated hemoglobin; TG: triglycerides; TC: total cholesterol; LDL: low density-lipoproteins; HDL: high density-lipoproteins.

**Table 4:** Changes in lipids and glucose homeostasis markers in women who had physical activity below recommendations (*n* = 5) and in women who exercised at least three times a week (*n* = 4).

	Group	Baseline mean ± SD	Observed change mean ± SD	Adjusted change^a^ mean ± SD	Adjusted effect^b^	
					Mean; CI	Inference^c^
Irisin (ng·ml^−1^)	Very active	15.32 ± 5.30	15 ± 29%	15 ± 28%	−31% −54 to 3%	Large^↓*∗∗*^
	Less active	14.34 ± 1.59	−18 ± 31%	−20 ± 13%		
Glucose (mg·dl^−1^)	Very active	77.25 ± 3.69	3 ± 3%	3 ± 4%	−2 % −10 to 6%	Small
	Less active	83.60 ± 1.95	1 ± 3%	1 ± 3%		
HbA1c (%)	Very active	4.53 ± 0.19	2 ± 5%	−1 ± 5%	4% −7 to 16%	Moderate
	Less active	4.86 ± 0.25	2 ± 3%	3 ± 3%		
Insulin (*µ*lU·ml^−1^)	Very active	3.58 ± 2.28	8 ± 66%	15 ± 80%	−20% −71 to 122%	Small
	Less active	2.38 ± 0.88	47 ± 37%	43 ± 37%		
TG (mg·dl^−1^)	Very active	131.80 ± 42.75	45 ± 24%	42 ± 28%	14% −22 to 65%	Small
	Less active	112.00 ± 28.65	59 ± 26%	61 ± 30%		
TC (mg·dl^−1^)	Very active	218.00 ± 45.85	19 ± 5%	18 ± 5%	−0.4% −8 to 8%	Trivial
	Less active	244.80 ± 55.39	14 ± 15%	18 ± 5%		
LDL (mg·dl^−1^)	Very active	109.75 ± 40.60	33 ± 14%	29 ± 8%	−3% −15 to 11%	Trivial
	Less active	129.20 ± 39.56	22 ± 18%	25 ± 12%		
HDL (mg·dl^−1^)	Very active	85.50 ± 10.28	−3 ± 5%	−3 ± 6%	−6% −29 to 24%	Small
	Less active	90.20 ± 23.59	−10 ± 30%	9 ± 29%		

*Note*. Less active: exercising less than three times a week, very active: exercising at least three times a week, and CI: 90% confidence interval. All data are percentages, with the exception of baseline values expressed in measurement units. Inferences shown in italic are clear at the 90% level of confidence. ^a^Adjusted to overall mean of the less active and very active groups at baseline. ^b^Adjusted mean change in the less active group minus adjusted mean change in the very active group. ^c^Magnitude thresholds (for difference in means divided by SD of control group): <0.20, trivial; 0.20–0.59, small; 0.60–1.19, moderate; 1.20–2.19, large; 2.2–4.0, very large. ^↑^Increase; ^↓^decrease. Asterisks indicate effects clear at the 5% level and likelihood that the true effect is substantial: ^*∗*^possible, ^*∗∗*^likely, ^*∗∗∗*^very likely, and ^*∗∗∗∗*^most likely.
